# Supplementary material for: Structural basis of transcription inhibition by the DNA mimic protein Ocr of bacteriophage T7
Source: eLife. 2020 Feb 10;9:e52125. doi: 10.7554/eLife.52125 (PMC7064336; doi:10.7554/eLife.52125)
Supplement: Supplementary file 1. [file elife-52125-supp1.docx]

**Supplementary File 1.** **Pairwise comparisons of *E. coli* cells expressing Ocr (WT/pBAD18cm[*ocr*]) *versus* cells not expressing Ocr (WT/pBAD18cm).**

| Growth Conditions | | | **Growth**  **Inhibition** |
| --- | --- | --- | --- |
| **Growth Medium** | Temperature | Ocr Induction |  |
| **LB broth (low salt)** | 37^o^C | - | NO |
| **LB broth (low salt)** | 37^o^C | 0.02%(^w^/_v_) arabinose | NO |
| **LB broth (low salt)** | 37^o^C | 0.20%(^w^/_v_) arabinose | NO |
| **LB broth (low salt)** | 25^o^C | - | NO |
| **LB broth (low salt)** | 25^o^C | 0.02%(^w^/_v_) arabinose | NO |
| **LB broth (low salt)** | 25^o^C | 0.20%(^w^/_v_) arabinose | NO |
| **LB broth (high salt)** | 37^o^C | - | NO |
| **LB broth (high salt)** | 37^o^C | 0.02%(^w^/_v_) arabinose | NO |
| **LB broth (high salt)** | 37^o^C | 0.20%(^w^/_v_) arabinose | NO |
| **LB broth (high salt)** | 25^o^C | - | NO |
| **LB broth (high salt)** | 25^o^C | 0.02%(^w^/_v_) arabinose | NO |
| **LB broth (high salt)** | 25^o^C | 0.20%(^w^/_v_) arabinose | NO |
| **Nutrient broth** | 37^o^C | - | NO |
| **Nutrient broth** | 37^o^C | 0.02%(^w^/_v_) arabinose | NO |
| **Nutrient broth** | 37^o^C | 0.20%(^w^/_v_) arabinose | NO |
| **Nutrient broth** | 25^o^C | - | NO |
| **Nutrient broth** | 25^o^C | 0.02%(^w^/_v_) arabinose | NO |
| **Nutrient broth** | 25^o^C | 0.20%(^w^/_v_) arabinose | NO |
| **Modified M9 medium** | 37^o^C | - | YES |
| **Modified M9 medium** | 37^o^C | 0.02%(^w^/_v_) arabinose | YES |
| **Modified M9 medium** | 37^o^C | 0.20%(^w^/_v_) arabinose | YES |
| **Modified M9 medium** | 25^o^C | - | YES |
| **Modified M9 medium** | 25^o^C | 0.02%(^w^/_v_) arabinose | YES |
| **Modified M9 medium** | 25^o^C | 0.20%(^w^/_v_) arabinose | YES |
